# Prognosis and prediction of antibiotic benefit in adults with clinically diagnosed acute rhinosinusitis: an individual participant data meta-analysis

**DOI:** 10.1186/s41512-023-00154-0

**Published:** 2023-09-05

**Authors:** Jeroen Hoogland, Toshihiko Takada, Maarten van Smeden, Maroeska M. Rovers, An I. de Sutter, Daniel Merenstein, Laurent Kaiser, Helena Liira, Paul Little, Heiner C. Bucher, Karel G. M. Moons, Johannes B. Reitsma, Roderick P. Venekamp

**Affiliations:** 1 Julius Center for Health Sciences and Primary Care, University Medical Center Utrecht, Utrecht University, Utrecht, The Netherlands; 2 Department of Epidemiology and Data Science, Amsterdam University Medical Centres, Amsterdam University, Amsterdam, The Netherlands; 3 Department of General Medicine, Shirakawa Satellite for Teaching And Research (STAR), Fukushima Medical University, Fukushima, Japan; 4 Radboud Institute for Health Sciences (RIHS), Radboud University Medical Center, Nijmegen, The Netherlands; 5 Department of Public Health and Primary Care, Ghent University, Ghent, Belgium; 6 Department of Family Medicine, Georgetown University Medical Center, Washington, DC, USA; 7 Department of Medicine, Division of Infectious Diseases, University Hospital Geneva, Geneva, Switzerland; 8 Department of General Practice, School of Primary, Aboriginal and Rural Health Care, University of Western Australia, Perth, Australia; 9 Department of General Practice and Primary Care, University of Helsinki, Helsinki, Finland; 10 Primary Care & Population Sciences Unit, Aldermoor Health Centre, University of Southampton, Southampton, UK; 11 Division of Clinical Epidemiology, Department of Clinical Research, University Hospital Basel and University of Basel, Basel, Switzerland

**Keywords:** Individual participant data meta-analysis, Randomized controlled trial, Acute rhinosinusitis, Antibiotic treatment, Individualized treatment effect, Prediction

## Abstract

**Background:**

A previous individual participant data meta-analysis (IPD-MA) of antibiotics for adults with clinically diagnosed acute rhinosinusitis (ARS) showed a marginal overall effect of antibiotics, but was unable to identify patients that are most likely to benefit from antibiotics when applying conventional (i.e. univariable or one-variable-at-a-time) subgroup analysis. We updated the systematic review and investigated whether multivariable prediction of patient-level prognosis and antibiotic treatment effect may lead to more tailored treatment assignment in adults presenting to primary care with ARS.

**Methods:**

An IPD-MA of nine double-blind placebo-controlled trials of antibiotic treatment (*n*=2539) was conducted, with the probability of being cured at 8–15 days as the primary outcome. A logistic mixed effects model was developed to predict the probability of being cured based on demographic characteristics, signs and symptoms, and antibiotic treatment assignment. Predictive performance was quantified based on internal-external cross-validation in terms of calibration and discrimination performance, overall model fit, and the accuracy of individual predictions.

**Results:**

Results indicate that the prognosis with respect to risk of cure could not be reliably predicted (c-statistic 0.58 and Brier score 0.24). Similarly, patient-level treatment effect predictions did not reliably distinguish between those that did and did not benefit from antibiotics (c-for-benefit 0.50).

**Conclusions:**

In conclusion, multivariable prediction based on patient demographics and common signs and symptoms did not reliably predict the patient-level probability of cure and antibiotic effect in this IPD-MA. Therefore, these characteristics cannot be expected to reliably distinguish those that do and do not benefit from antibiotics in adults presenting to primary care with ARS.

**Supplementary Information:**

The online version contains supplementary material available at 10.1186/s41512-023-00154-0.

## Background

Acute rhinosinusitis (ARS) is one of the conditions with highest antibiotic over-prescription rates in adults [1, 2]. With antimicrobial resistance posing a serious threat to global public health [3], continuous efforts are needed to reduce inappropriate antibiotic prescription in primary care [4]. One of the reasons for the persistent habit of general practitioners (GPs) to prescribe antibiotics might be attributed to their clinical impression that there is a subgroup of patients with clinically diagnosed ARS that actually do benefit from antibiotics [5]. There is also some evidence to substantiate this impression; antibiotics seem to have larger effects in those with radiologically confirmed ARS, in particular those with a fluid level or total opacification in any sinus on computed tomography [6]. Previous attempts to identify these subgroups based on common signs and symptoms were not successful, including an individual patient data meta-analysis (IPD-MA) of randomized controlled trials (RCTs) comparing antibiotics with placebo in adults with clinically diagnosed ARS [7]. This preceding IPD-MA applied conventional (univariable) subgroup analysis in which potential effect modification of single signs and symptoms was assessed one at the time. This approach does not focus on the absolute risk scale that is of most interest for clinical decision making (instead focusing on relative effects), likely under-represents underlying clinical heterogeneity (individuals may vary in more than one relevant aspect) [8, 9], and is known to be statistically inefficient [10]. Multivariable risk prediction modelling allowing for simultaneous analysis of multiple baseline variables that may influence treatment effect has the potential to overcome these problems [9, 11–14]. Such a model provides patient-level outcome risk predictions for both treatment assignments and hence also predicts the patient-level absolute benefit of antibiotic treatment of interest. Due to the required sample size, IPD from multiple studies provide a good source for model development [15, 16]. Subsequently if accurate predictions can be made, they can inform treatment decisions in clinical practice, informing on the probability of fast spontaneous resolution of symptoms and the anticipated benefit of antibiotic treatment at the patient-level. With this aim, we applied multivariable prediction modelling methods to IPD of multiple RCTs comparing antibiotics with placebo in adults with clinically diagnosed ARS.

## Methods

The protocol of this IPD-MA has been registered in PROSPERO (registration number CRD 42020220108) and published [17]. A detailed description of the rationale and methodology can be found in the protocol publication [17]. We followed recommendations provided in the Predictive Approaches to Treatment effect Heterogeneity (PATH) statement [12], guidance on the individualized treatment effect prediction [14], and guidance on the use of IPD-MA of diagnostic and prognostic modelling studies [16], and reported according to the TRIPOD [18, 19] and PRISMA-IPD statement [20].

### Study identification and selection

We conducted a systematic search to identify eligible studies. First, the reference list of the 2018 Cochrane review on antibiotics for ARS in adults [6] was reviewed for any relevant studies published since the 2008 IPD-MA [7]. Next, we updated the systematic electronic searches of the Cochrane review (online supplementary Table S1) from January 18, 2018 (date of last search), to September 1, 2020, to increase the yield of potentially relevant trials. No language restrictions were applied.

Titles and abstracts of the unique records retrieved from these electronic databases were screened and the full text of all potentially eligible articles was reviewed against the following predefined criteria: (i) RCT comparing antibiotics with placebo and (ii) enrolled adults ($\ge 16$ years) presenting to primary care with uncomplicated ARS based on clinical signs and symptoms. Studies involving children (<16 year), referred patients, hospitalized patients, and those involving highly specialized populations (e.g. those with immunodeficiency, odontogenic sinusitis, or malignancy) were excluded. In addition, reference lists of all eligible studies as well as those from relevant systematic reviews were screened for any further potential studies and contributing review authors were asked if they knew any additional (published or unpublished) studies. Study authors of eligible trials were contacted and invited to provide the de-identified, complete dataset of their original trial.

### Quality assessment of included studies

Methodological quality of the included studies was assessed using the Cochrane Risk of Bias 2 tool [21]. If information regarding study quality was unclear or undisclosed, individual trial authors were contacted to provide further clarification.

### Outcome assessment

All retrieved IPD were assembled in a single dataset. The predefined outcome of interest was cure at 8–15 days (yes vs no) [17], which was available in all studies.

### Candidate predictors

Candidate predictors were selected based on clinical reasoning, knowledge from existing literature, and availability in the IPD set. Next to (i) treatment assignment (oral antibiotics vs placebo) which was available in all trials, the following pre-specified candidate predictors of treatment effect were available in at least 50% of studies: (ii) sex, (iii) age (in years), (iv) preceding upper respiratory tract infection (URTI), (v) symptom duration prior to enrolment (in days), (vi) pain on bending, (vii) teeth pain, (viii) unilateral facial pain, (ix) self-reported purulent nasal discharge (PNDsr), (x) symptom severity, (xi) presence of fever ($>37.5$ C; yes vs no), (xii) purulent nasal discharge upon examination (PNDex), and (xiii) purulent pharyngeal discharge upon examination (PPDex). For symptom severity, we used the standardized 0–100 severity as used in the 2008 IPD-MA [7] which was based on a (scaled) logistic transformation of the severity measures applied in the individual trials. The following pre-specified candidate predictors [17] could not be included in our analysis due to not being measured in $>50$% of trials: previous ARS, anosmia, cacosmia, double sickening, overall clinical impression, C-reactive protein (CRP), and erythrocyte sedimentation rate (ESR) values. The available set of candidate predictors was assessed for both prognostic value and for differential treatment effect with respect to cure at 8–15 days; see the ‘Statistical analysis’ section for further details.

### Sample size considerations

We calculated the maximum number of candidate predictors based on an anticipated number of 2500 patients in the IPD set, with an average outcome prevalence of 60% cure, and a desired 0.05 accuracy in terms of mean absolute prediction error [22]. Since the available guidance does not yet extend to clustered IPD, we conservatively estimated our effective sample size to be 1250 which allows for evaluation of 25 parameters in the model based on a presumed Cox-Snell $R^2$ of 0.175, which is also expected to keep shrinkage below 10% and the expected Cox-Snell $R^2$ within 5%.

### Statistical analysis

#### Handling of missing data

Missing data were imputed using a fully Bayesian joint modelling approach [23]. A total of 50 imputations were derived as compatible with a generalized linear mixed effects analysis model with a logistic link function, random intercepts per study, main effects for treatment and each of the candidate predictors, and treatment-predictor interaction terms [24]. All effect were modelled to be linear on the linear predictor scale since spline-based exploratory analysis based on the complete cases did not indicate clear non-linear predictor-outcome relations.

#### Descriptive statistics

First, predictors and outcome distributions were summarized in each study. Next, a multinomial membership model was used to evaluate multivariable between-study heterogeneity in predictor and outcome distributions [25]. Such a membership model predicts study membership based on the candidate predictors and outcome and hence illustrates the degree to which multivariable differences between studies allow a model to predict to which study an individual belongs. Details are provided in the online supplementary material 1.

#### Main analysis: prediction model development

In the primary analysis, all available candidate predictors and treatment assignment were included as main effects in a logistic mixed effects regression model with random intercepts per study [17]. The requirement for a random main treatment effect was also evaluated. Symptom duration was heavily skewed to the right and therefore log-transformed. Due to between-study variability in outcome assessment, study level variables ‘number of days between baseline and outcome measurement’ and ‘type of outcome measurement’ were added to the model. To explore treatment effect heterogeneity, all treatment-predictor interactions were added to the model. In line with the study protocol [17], this extended model was compared to the main-effects-only model by means of a likelihood ratio test (based on the $D_3$-statistic [24]), hence testing the joint contribution of all treatment-predictor interactions against the null hypothesis that all interaction parameters are zero. The main purpose was to avoid extensive data-driven search of interactions in the main analysis and select either all or none of the treatment-predictor interactions.

In mathematical notation, the complete model for the Bernoulli distributed outcome cure, for individual *i* from the study *j*, can be written as

$$\begin{aligned} \textrm{Cure}_{i}\sim & {} \textrm{Bernoulli}(\textrm{prob}_{\textrm{Cure} = 1} = \widehat{P}) \\ \log \left( \frac{\hat{P}}{1 - \hat{P}}\right)= & {} (\beta _0 + b_{0j}) + (\beta _{1} + b_{1j})\textrm{Treat}_{\textrm{1i}} + \varvec{\beta }'_{\textrm{main}} \varvec{x}_i + \varvec{\gamma }'_{\textrm{int}} \varvec{x}_i \textrm{Treat}_{\textrm{1i}} \\ \left( \begin{array}{c} b_{0j} \\ b_{1j} \end{array} \right)\sim & {} N \left( \left( \begin{array}{c} 0 \\ \\ 0 \end{array} \right) , \left( \begin{array}{cc} \sigma ^2_{b_{0j}} &{} \rho _{b_{0j} b_{1j}} \\ \rho _{b_{1j}b_{0j}} &{} \sigma ^2_{b_{1j}} \end{array} \right) \right) \text {, for Trial j = 1,} \dots \text {,J} \end{aligned}$$

where $\beta _0$ denotes the overall intercept, $\beta _1$ the main treatment effect, $\varvec{\beta }_{\textrm{main}}$ the vector of main effect coefficients for each of the candidate predictors in $\varvec{x}_i$, $\varvec{\gamma }_{\textrm{int}}$ the vector of corresponding treatment-predictor interactions, and $b_{0}$ and $b_{1}$ denote the random intercepts and treatment effects. Hence, the pre-defined likelihood ratio test for the combined treatment-predictor interactions tests against the null hypothesis that $\varvec{\gamma }_{\textrm{int}}=\varvec{0}$. In addition to this global test, the exploratory analysis described in the next section did search for individual interactions. Note that in the absence of treatment-predictor interactions (also known as predictive effects [26]), the model reduces to a prognostic model [27] with the addition of a main treatment effect $\beta _1$ that may vary across studies according to $b_{1}$.

#### Secondary and exploratory analyses

As opposed to the study of individual treatment interactions, baseline risk-modelling [12] was pre-specified as a secondary analysis [17]. This approach entails an evaluation of possible treatment effect heterogeneity as a function of baseline risk-model and has been recommended in settings where (i) an overall treatment effect is well established, (ii) several large RCTs are available for analysis, and (iii) when substantial identifiable heterogeneity of outcome risk in the trial population(s) is anticipated [12]. In addition, in order to evaluate the possible benefit of model simplification in terms of generalizability, model reduction was evaluated using a relaxed-lasso procedure in exploratory analysis [28, 29]. The relaxed-lasso was performed on stacked imputed data [30], with fixed and unpenalized study intercepts, an unpenalized main treatment effect, and penalized main effects for all candidate predictors, and penalized interactions between all candidate predictors and treatment. Tuning parameters lambda (degree of penalization selection) and gamma (degree of post-selection relaxation) selected according to the 1 standard error rule based on 10-fold cross-validation.

#### Evaluation of prediction model performance

Prediction model performance with respect to the prediction of outcome risk and absolute antibiotic treatment effect was evaluated by means of calibration performance (extent of agreement between predicted risk and observed events), discrimination performance (with the aim to quantify whether predicted risk correctly rank-orders actual risk), Nagelkerke $R^2$ (as a measure of overall model fit), and Brier score (as a measure of prediction accuracy). Performance was assessed using internal-external cross-validation (IECV) [31]. Standard errors for each of the measures were derived based on 500 bootstrap samples. Meta-analysis was used to summarize the main IECV results using restricted maximum likelihood-based estimates of between study variability, inverse variance weighting, and Hartung and Knapp adjustment [32]. Prediction model performance with respect to predicted absolute antibiotic treatment effect (i.e. on the risk difference level) was evaluated in terms of discriminative performance using the c-for-benefit [33] and in terms of calibration in the form of predicted versus observed treatment effect in quartiles of predicted treatment effect.

## Results

### Study inclusion and study characteristics

**Fig. 1:**
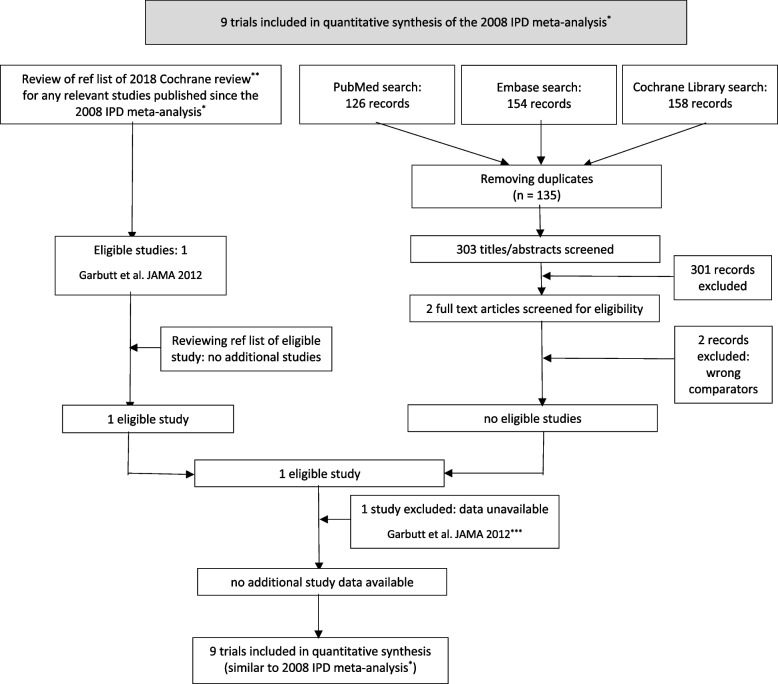
Inclusion flowchart. * refers to Young et al. [7], ** refers to Lemiengre et al. [6], and *** refers to Garbutt et al. [43]

The 2008 IPD-MA [7] included data from 9 trials [34–42]. An additional eligible study [43] was identified from reviewing the reference list of the 2018 Cochrane review [6]. This study with 166 participants (online supplementary Table S2) was excluded since authors were not able to provide IPD. No further eligible studies were found after screening the 303 unique records retrieved from the electronic database searches or through additional routes (Fig. 1). This left 9 trials with 2539 participants aged ($\ge 16$ years) for inclusion [34–42]. Details on the design characteristics of the included studies are shown in online supplementary Table S3. All studies were double-blind, placebo controlled randomized trials and conducted in high-income countries in Europe and in the US. One trial used a 2×2 factorial design [41], and data were split into two sub-trials: antibiotics vs. placebo without concomitant nasal steroids in both groups (Williamson1) or antibiotics vs. placebo with concomitant nasal steroids in both groups (Williamson2). Participants from the intervention groups received beta-lactam antibiotics (mainly amoxicillin, but also amoxicillin clavulanate or phenoxymethylpenicillin), macrolides (azithromycin), or tetracyclines (doxycycline). Sample size of the included trials ranged from 135 to 503.

### Quality assessment of included studies

The quality assessment of included studies is summarized in online supplementary Fig. S1. The risk of bias could not be assessed for the unpublished Schering-Plough trial [42]. Overall risk of bias was judged low for the other included studies.

### Missing data

The percentage of missing data varied greatly across studies and variables (online supplementary Table S4). Including both sporadic (i.e. partly, but not completely missing in a certain study) and systematically missing data (i.e. completely missing in a certain study), the percentage of missingness was below 10% for all variables except for preceding URTI (66%, unavailable in 5/10 studies) pain on bending (62%, unavailable in 5/10 trials), pain in teeth (56%, unavailable in 4/10 trials), unilateral facial pain (41%, unavailable in 2/10 trials), and PPDex (52%, unavailable in 5/10 trials).

### Descriptive statistics

Descriptive statistics for each of the trials after imputation of missing data are shown in Table 1 and visually presented in online supplementary Fig. S2. Studies differed with respect to both outcome occurrence (range 35–77%) and the prevalence of predictors of interest. Most notably, symptom duration prior to enrolment and the prevalence of pain on bending, PNDsr, and PPDex varied substantially across studies.

**Table 1:** Descriptive statistics for each of the ten trials after multiple imputation

Trial	Bucher	De Sutter	Kaiser	Meltzer	Merenstein	Schering-Plough	Stalman	Varonen	Williamson1	Williamson2
Number of participants	251	368	266	490	135	458	191	150	116	114
Antibiotic assignment (%)	49	49	51	50	50	48	51	59	51	45
Female (%)	54	57	52	66	69	70	64	70	72	76
Age in years, mean (SD)	36.6 (13.1)	38.4 (14.2)	32.8 (11.6)	35.7 (12.2)	33.9 (9.8)	38.7 (14.6)	37.0 (11.4)	39.7 (13.4)	43.3 (15.5)	43.8 (12.7)
Preceding URTI (%)	56	79	35	78	52	76	92	56	71	67
Symptom duration in days median (IQR)	4 (3,7)	6 (3,10)	4 (3,7)	13 (10,19)	9 (7-14)	13 (10,19)	7 (6,14)	5 (3,9)	5 (3,10)	5 (3,7)
Pain on bending (%)	82	69	27	86	81	85	90	78	82	85
Pain in teeth (%)	24	44	14	85	28	81	50	19	37	56
Unilateral facial pain (%)	54	55	14	98	39	98	42	23	52	51
PNDsr (%)	98	88	51	67	84	53	83	48	74	67
Symptom severity,	44.2	50.6	46.0	41.8	43.7	48.5	48.1	55.7	56.2	48.5
median (IQR)	(19.8, 67.8)	(24.4, 62.7)	(15.8, 74.1)	(23.4, 79.6)	(27.0, 78.6)	(28.4, 77.5)	(23.1, 74.7)	(25.7, 76.0)	(27.7, 82.1)	(27.7, 73.1)
Fever (%)	14	7	11	1	1	2	4	18	3	4
PNDex (%)	66	55	33	33	32	31	12	32	39	31
PPDex (%)	21	45	18	45	55	34	9	72	22	25
Cure (%)	69	35	56	69	49	66	65	77	64	65

*IQR* Interquartile range, *PNDex* Purulent nasal discharge upon examination, *PNDsr* Purulent nasal discharge self-reported, *PPDex* Purulent pharyngeal discharge upon examination, *SD* Standard deviation, *URTI* Upper respiratory tract infection. Grey figures relate to systematically missing data

Online supplementary Table S5 further illustrates the between-study heterogeneity. The membership model had high discriminative ability for all studies, indicating substantial differences in predictor and outcome distributions across studies. Based on a common intercept and common predictor-outcome associations, the observed outcome incidence deviated somewhat from the expectation for four trials (Merenstein et al. [40], Kaiser et al. [35], de Sutter et al. [36], and Varonen et al. [38]), indicating that the observed incidence of cure could not be completely explained by the modelled effects of case-mix differences (online supplementary Fig. S3).

### Main analysis results

Estimates for the pre-specified main effects model are shown in Table 2. The pre-defined pooled likelihood ratio test of the combined treatment-predictor interactions was non-significant and they were not included in the model (D3 statistic 0.54, $df_1$ 12, $df_2$ 7497, *p* = 0.89). Significant patient-level associations with the risk of cure were found for antibiotic treatment (OR 1.34 [1.13 to 1.59]), age (OR 0.91 per 10 years [0.85 to 0.97]), log symptom duration prior to enrolment (OR 0.76 [0.65 to 0.89]), and symptom severity (OR 0.87 [0.82 to 0.91]). A significant study-level association with the risk of cure was found for outcome assessment based on clinical examination or a combination of methods vs. symptom diary (OR 0.40 [0.19 to 0.84]). Despite these main effect estimates, there was still considerable unexplained between-study variability in the outcome as shown in the random intercepts estimates (online supplementary Fig. S4). The estimated standard deviation of the random intercept distribution was 0.33, which has been referred to as ‘reasonable heterogeneity’ (Spiegelhalter et al. Section 5.7 [44]). The largest deviations from the overall mean were estimated for study data from Merenstein (−0.45), de Sutter (−0.44), Kaiser (0.44), and Varonen (0.48), corresponding to mean changes in modelled individual risks (i.e. the difference between modelled risk *with* the estimated random intercepts and with the random intercepts set to zero) of −10.5%, −10.1%, +10.6%, and +9.8%. The addition of a random main treatment effect to the model resulted in near-zero estimated variability ($\hat{\sigma }^2_{b_{1j}}$ = 0.001) with unidentifiable correlation between intercept and treatment effect variability; hence, the random treatment effect was dropped.

**Table 2:** Main effect estimates of prognostic factors for cure, based on the random intercept model as derived from IPD of ten trials. Coefficients (log(OR)), standard errors, odds ratios (OR), and 95% confidence intervals (CI) were pooled across imputations. The mean standard deviation of the random intercepts was 0.33

	$\hat{\beta }$	se	95% CI	OR (95% CI)
Intercept	0.83	0.88	(−0.89, 2.56)	2.30 (0.41, 12.98)
Antibiotics (yes)	0.29	0.09	(0.12, 0.46)	1.34 (1.13, 1.59)
Sex, female	−0.09	0.09	(−0.27, 0.09)	0.92 (0.76, 1.10)
Age, per 10 years	−0.10	0.03	(−0.16, −0.03)	0.91 (0.85, 0.97)
Preceding URTI	0.23	0.19	(−0.15, 0.61)	1.26 (0.86, 1.84)
Symptom duration in log(days)	−0.27	0.08	(−0.42, −0.12)	0.76 (0.65, 0.89)
Pain on bending	0.12	0.18	(−0.23, 0.47)	1.13(0.80, 1.60)
Pain in teeth	−0.12	0.15	(−0.42, 0.18)	0.89 (0.66, 1.20)
Unilateral facial pain	0.14	0.13	(−0.11, 0.40)	1.15 (0.89, 1.49)
PNDsr	0.17	0.11	(−0.05, 0.39)	1.19 (0.95, 1.48)
Symptom severity^a^	−0.14	0.03	(−0.20, −0.09)	0.87 (0.82, 0.91)
Fever	−0.25	0.19	(−0.63, 0.13)	0.78 (0.53, 1.14)
PNDex	0.07	0.10	(−0.12, 0.26)	1.07 (0.89, 1.29)
PPDex	−0.19	0.15	(−0.48, 0.10)	0.82 (0.62, 1.10)
Time to outcome measurement (days)	0.03	0.08	(−0.12, 0.18)	1.03 (0.89, 1.20)
Outcome type: other, (ref. diary)	−0.92	0.38	(−1.66, −0.18)	0.40 (0.19, 0.84)
Outcome type: telephone, (ref. diary)	−0.13	0.37	(−0.85, 0.59)	0.88 (0.43, 1.81)

*PNDex* Purulent nasal discharge upon examination, *PNDsr* Purulent nasal discharge self-reported, *PPDex* Purulent pharyngeal discharge upon examination, *URTI* Upper respiratory tract infection

^a^Per point on the inverse logit transformation of (severity score / 100)

IECV performance estimates indicated poor prediction performance and overall model fit of the main effects model (Table 3 and online supplementary Fig. S5). The pooled IECV c-statistic estimate (0.58) did indicate some discriminative ability with a prediction interval (PI) of 0.56–0.62. However, while $R^2$ and Brier scores were heterogeneous across studies, their pooled estimates clearly indicate poor performance with $R^2$ −0.08 (PI −0.48, 0.32) and Brier score 0.24 (PI 0.15, 0.34). It is worth noting that, in contrast to the c-statistic, both $R^2$ and Brier score depend on accurate intercept estimates and will therefore reflect the unexplained between-study variability associated with the random intercepts. Both measures indicate that the main effects model did not provide accurate absolute risk predictions for the hold-out studies. This lack of generalizability between studies was further illustrated by the large prediction intervals for the estimated calibration intercepts [−1.06 and 1.11] and calibration slopes [0.18 and 1.38]. While these intervals include the favourable values of 0 and 1, they also include a large range of unfavourable calibration estimates.

**Table 3:** IECV results for risk (of cure) prediction based on the main effects model as derived from IPD of ten trials

	C-statistic	R2	Brier	Intercept	Slope
Bucher	0.59	0.02	0.21	0.08	0.79
De Sutter	0.55	−0.49	0.30	−0.82	0.38
Kaiser	0.55	−0.60	0.33	0.61	0.38
Meltzer	0.62	0.03	0.21	0.00	1.57
Merenstein	0.57	−0.26	0.29	−0.51	0.57
Schering-Plough	0.58	0.02	0.22	0.15	0.88
Stalman	0.59	0.04	0.22	−0.04	0.92
Varonen	0.62	−0.27	0.21	0.94	1.09
Williamson1	0.60	0.01	0.23	0.09	0.67
Williamson2	0.62	0.04	0.22	−0.10	1.04

*IECV* Internal-external cross-validation

As a sensitivity analysis, all analyses were re-run after omitting data from the Schering-Plough study [42], as the risk of bias could not be assessed for this trial. This, however, did not substantially change model performance (online supplementary Table S6). In summary, the absolute risk of cure could not be reliably predicted based on the available predictors and can hence not be used to differentiate between low-risk and high-risk individuals to inform treatment decisions.

### Secondary and exploratory analyses results

Given the lack of reliable risk predictions based on the main risk model, further modelling using these predictions as inputs was not deemed relevant. Therefore, baseline risk-modelling, which essentially evaluates outcome risk modification by treatment, was not performed. As anticipated based on previous findings, the exploratory relaxed-lasso procedure led to substantial model reduction: only a main effect for symptom severity and unpenalized parameters (study intercepts and treatment assignment) were left in the model.

Contrary to the large between-study heterogeneity in terms of model performance as observed in the main analysis, evaluation of the marginal relative treatment effect (OR 1.32; 95% CI 1.11–1.56) did not reveal any between-study heterogeneity (not shown), confirming earlier results [7].

### Evaluations of absolute treatment effect prediction

To supplement outcome risk evaluations, individual predictions of absolute treatment effect were evaluated (online supplementary Fig. S6). The IECV estimate for discriminative performance (c-for-benefit) was 0.50 for the main effects model, indicating absence of discriminative ability. Therefore, further examination of calibration performance was not deemed relevant.

## Discussion

This large IPD-MA of high-quality antibiotic therapy trials in adults presenting to primary care with clinically diagnosed uncomplicated ARS evaluated patient-level variability in prognosis and antibiotic treatment effect. Such variability could not be reliably predicted based on demographics and clinical signs and symptoms, illustrating that these characteristics do not contribute to the identification of patients that are most likely to benefit from antibiotics.

In more detail, meaningful discrimination between patients with respect to treatment effect could have been based on (i) important treatment-predictor interactions (i.e. genuine treatment effect heterogeneity) or (ii) a treatment effect with a constant odds ratio in combination with accurate and meaningful variability in prognosis [14]. The main results did identify several prognostic factors [27], with increasing age, symptom duration, and symptom severity decreasing the probability of cure at 8–15 days. In line, the model had some discriminative ability with respect to prognosis (IECV-based c-statistic 0.58), which reflects some degree of correct rank-ordering with respect to predicted risk. However, the remaining degree of uncertainty was too large for these effects to translate into reliable absolute risk estimates as needed to guide patient management.

A strong aspect of this study was the large sample size derived from multiple high-quality trials. This allowed for careful handling of missing data and consistent multivariable prediction modelling of antibiotic treatment effect across studies [16]. The lack of predictable between-subject heterogeneity of antibiotic benefit was robust, since our conservative primary analysis’ findings were supported by those derived from exploratory relaxed-lasso modelling.

Several limitations deserve further attention. First, we observed a high degree of heterogeneity across studies, in particular with respect to the outcome definition, outcome assessment, and studied populations. In terms of outcome definition and assessment, this was alleviated by adjustment for study level information on time to outcome assessment and type of outcome assessment. With respect to heterogeneity in study populations, internal-external cross-validation revealed that a common model did not describe the data well. Second, we did not have sufficient information to include time-to-cure instead of the available dichotomous outcome data, which would likely be a more sensitive outcome. Also, severely unwell individuals with prolonged illness duration may be underrepresented in the included trials, and the modelled relationships between predictors and outcome may not generalize to the wider population presenting in primary care. Third, there was a substantial amount of systemically missing data. Although carefully handled using multiple imputation, this still represents loss of information which likely has influenced our results (e.g. possibly weakening predictor-outcome associations). Finally, potential important signs (severe pain, double sickening) and laboratory findings (CRP, ESR) were not available in a sufficient number of trials. It is, however, uncertain whether the availability of these variables would have impacted our findings. For example, CRP was found to be of value in a recent diagnostic IPD-MA for ruling out, but not for ruling in target conditions associated with antibiotic benefit in adults suspected of ARS [45]. A recent review of diagnostic accuracy studies of CRP, ESR, white blood cell counts, procalcitonin, and nasal nitric oxide for detecting acute bacterial rhinosinusitis (ABRS) found that especially elevated CRP and ESR are associated with higher probability of ABRS. However, CRP and ESR were still found insufficiently accurate for predicting ABRS [46]. Further research in this field should focus on the added value of novel point-of-care tests or novel devices such as those aimed at gaining specimens from draining sinuses [47] over readily available signs and symptoms such as age, symptom duration, and severity. Early-stage investigations of biomarker combination tests as well as host gene expression diagnostics suggest that these point-of-care tests have the potential to discriminate between viral and bacterial aetiology of RTI, but high-quality prospective clinical validation studies in primary care are needed to confirm their potential [48–50].

Lastly, some discussion with respect to the choice of modeling is warranted. In principle, all model parameters could vary across studies, but the limited number of studies did not provide sufficient information to thoroughly estimate such variability. Therefore, in line with Seo et al. [51], we assumed the main predictor effects and treatment-predictor interactions to be common across studies (fixed). On the contrary, when interest is primarily in a small number of parameters relating to *relative* treatment effect (hence treating other parameters as nuisance parameters), other approaches are available [52]. Similar arguments hold for the analysis of isolated prognostic factors [53]. In our case, all model parameters were of interest, with the fixed effects revealing common patterns across studies. These common patterns are exactly the patterns of interest for generalizable prediction accuracy, but do not provide a detailed description of (unexplained) between-study variability.

In conclusion, this IPD-MA using demographics and signs and symptoms did not result in reliable patient-level predictions of either prognosis or antibiotic treatment effect in adults presenting to primary care with clinically diagnosed ARS. While future research may reveal markers that aid the identification of adults with clinically diagnosed ARS most likely to benefit from antibiotics, current evidence does not support individualized treatment selection in adults with uncomplicated ARS.

### Supplementary information

**Additional file 1.** Online supplementary material. Word file *ARS_Abx_IPDMA_supplementary_DAPR.doc* contains the online supplementary material.

## Data Availability

The individual patient data for the included trials is not publicly available due to privacy regulations.

## Abbreviations

ABRS: Acute bacterial rhinosinusitis
ARS: Acute rhinosinusitis
CRP: C-reactive protein
ESR: Erythrocyte sedimentation rate
IECV: Internal-external cross-validation
IPD-MA: Individual patient data meta-analysis
PPDex: Purulent pharyngeal discharge upon examination
PNDex: Purulent nasal discharge upon examination
PNDsr: Self-reported purulent nasal discharge
URTI: Upper respiratory tract infection
