# Supplementary material for: Prognosis and prediction of antibiotic benefit in adults with clinically diagnosed acute rhinosinusitis: an individual participant data meta-analysis
Source: Diagn Progn Res. 2023 Sep 5;7:16. doi: 10.1186/s41512-023-00154-0 (PMC10478354; doi:10.1186/s41512-023-00154-0)
Supplement: Supplementary file 1 — Additional file 1. Online supplementary material. Word file ARS_Abx_IPDMA_supplementary_DAPR.doc contains the online supplementary material. [file 41512_2023_154_MOESM1_ESM.docx]

**SUPPLEMENTARY MATERIAL**

**Prognosis and antibiotic benefit prediction in adults with clinically diagnosed acute rhinosinusitis: an individual patient data meta-analysis**

**Content**

**Table S1. Search strategy**

**Table S2. Characteristics of excluded studies**

**Table S3. Characteristics of included studies**

**Table S4. Percentage of missing values**

**Table S5. Between-study heterogeneity in predictor and outcome distributions**

**Table S6. IECV results for the main effects model after omission of Schering-Plough data**

**Figure S1. Risk of bias assessment**

**Figure S2. Visual representation of the descriptive statistics across studies**

**Figure S3. Expected versus observed percentage cure in the context of the study of case-mix heterogeneity between studies**

**Figure S4. Boxplots of the distribution of random intercept estimates for each of the ten studies.**

**Figure S5. Internal-external cross-validation (IECV) of predicted of risk of cure (main analysis model)**

**Figure S6. Internal-external cross-validation (IECV) of predicted of individualized treatment effect (main analysis model)**

**Supplementary Material 1. Details of statistical analysis**

**References**

**Table S1. Search strategy**

| We searched the following databases from 18 January 2018 to 1 September 2020 using the database specific search strategies specified below: (1) MEDLINE via Ovid (from January 2018 to September 2020), (2) the Cochrane Central Register of Controlled Trials (CENTRAL; 2020, Issue 8) in the Cochrane Library, and (3) Embase via Ovid (from January 2018 to September 2020). | |
| --- | --- |
| **Database** | **Search strategy** |
| **Medline (Ovid)** | 1 exp Sinusitis/ (21250)  2 sinusit*.tw. (15723)  3 Rhinitis/ (13111)  4 rhinit*.tw. (27817)  5 rhinosinusit*.tw. (9659)  6 nasosinusit*.tw. (43)  7 ((suppurative or purulent) adj2 (nasal discharge or rhinitis or rhinorrhoea or rhinorrhoea)).tw. (258)  8 1 or 2 or 3 or 4 or 5 or 6 or 7 (58749)  9 exp Anti-Bacterial Agents/ (745280)  10 antibacterial*.tw. (80997)  11 antibiotic*.tw. (351647)  12 exp Amoxicillin/ (11514)  13 amoxicillin*.tw,nm. (21190)  14 Ampicillin/ (13596)  15 ampicillin*.tw,nm. (30363)  16 Azithromycin/ (5815)  17 azithromycin.tw,nm. (10586)  18 Cefaclor/ (838)  19 cefaclor.tw,nm. (1816)  20 exp Cefadroxil/ (474)  21 cefadroxil.tw,nm. (743)  22 cefatrizine.tw,nm. (118)  23 Cefuroxime/ (2220)  24 cefuroxim*.tw,nm. (5131)  25 cefuroxim*.tw,nm. (5131)  26 cephalexin*.tw,nm. (3687)  27 Cephalosporins/ (19613)  28 cephalosporin*.tw,nm. (34198)  29 Ciprofloxacin/ (13624)  30 ciprofloxacin*.tw,nm. (30022)  31 Clarithromycin/ (6413)  32 clarithromycin*.tw,nm. (11063)  33 clindamycin*.tw,nm. (12705)  34 Doxycycline/ (9972)  35 doxycyclin*.tw,nm. (17842)  36 Erythromycin/ (13875)  37 erythromycin*.tw,nm. (26744)  38 Fluoroquinolones/ (13739)  39 fluoroquinolone*.tw,nm. (24129)  40 levofloxacin.tw,nm. (8468)  41 Lincomycin/ (2146)  42 lincomycin*.tw,nm. (3557)  43 Macrolides/ (12776)  44 macrolide*.tw,nm. (24600)  45 Minocycline/ (6045)  46 minocyclin*.tw,nm. (9290)  47 Miocamycin/ (278)  48 (miocamycin* or miokamycin*).tw,nm. (313)  49 moxifloxacin*.tw,nm. (5312)  50 norfloxacin.tw,nm. (5300)  51 Norfloxacin/ (2496)  52 Ofloxacin/ (6055)  53 ofloxacin.tw,nm. (9865)  54 Penicillins/ (40419)  55 penicillin*.tw,nm. (82208)  56 Quinolones/ (11998)  57 quinolone*.tw,nm. (23464)  58 Spiramycin/ (718)  59 spiramycin.tw,nm. (1560)  60 telithromycin.tw,nm. (971)  61 tetracyclines/ or tetracycline/ (24207)  62 tetracycline*.tw,nm. (48153)  63 Trimethoprim Sulfamethoxazole Combination/ (7052)  64 trimethoprim sulfamethoxazole combination.tw,nm. (59)  65 cotrimoxazole*.tw,nm. (3309)  66 9 or 10 or 11 or 12 or 13 or 14 or 15 or 16 or 17 or 18 or 19 or 20 or 21 or 22 or 23 or 24 or 25 or 26 or 27 or 28 or 29 or 30 or 31 or 32 or 33 or 34 or 35 or 36 or 37 or 38 or 39 or 40 or 41 or 42 or 43 or 44 or 45 or 46 or 47 or 48 or 49 or 50 or 51 or 52 or 53 or 54 or 55 or 56 or 57 or 58 or 59 or 60 or 61 or 62 or 63 or 64 or 65 (992370)  67 8 and 66 (7037)  68 randomized controlled trial/ (527056)  69 Controlled Clinical Trial/ (94121)  70 (randomized or placebo or randomly or trial or groups).tw. (3084618)  71 Drug Therapy/ (30803)  72 68 or 69 or 70 or 71 (3245370)  73 Animals/ (6797267)  74 Humans/ (19166925)  75 73 not 74 (4779851)  76 72 not 75 (2784395)  77 67 and 76 (1237)  78 limit 77 to dt=20180118-20200930 (126) |
| **Cochrane Library** | #138 MeSH descriptor: [Sinusitis] explode all trees (1041)  #139 sinusit* (3194)  #140 MeSH descriptor: [Rhinitis] explode all trees (3955)  #141 (rhinit*):ti,ab,kw (10156)  #142 rhinosinusit* (1509)  #143 nasosinusit* (48)  #144 ((suppurative or purulent) NEAR/2 (nasal discharge or rhinitis or rhinorrhoea or rhinorrhoea)) (320)  #145 #138 or #139 or #140 or #141 or #142 or #143 or #144 (13593)  #146 MeSH descriptor: [Anti-Bacterial Agents] explode all trees (12299)  #147 (antibiotic*):ti,ab,kw (31994)  #148 Amoxicillin OR ampicillin OR azithromycin OR cefaclor OR cefadroxil OR cefatrizine OR cefuroxim* OR cephalexin* OR cephalosporin* OR ciprofloxacin* OR clarithromycin* OR clindamycin (18741)  #149 doxycyclin OR erythromycin* OR fluoroquinolone* (4373)  #150 levofloxacin* OR lincomycin* OR macrolide* OR minocyclin* OR miocamycin* (4280)  #151 miokamycin* OR moxifloxacin* OR norfloxacin* OR ofloxacin* OR penicillin* (7410)  #152 quinolone* OR spiramycin OR telithromycin OR tetracyclin* OR trimethoprim* OR cotrimoxazol* (6739)  #153 #146 or #147 or #148 or #149 or #150 or #151 or #152 (54772)  #154 #153 and #145 (1351)  #155 limit CENTRAL to 2018-2020 (158) |
| **Embase (Ovid)** | 1 exp sinusitis/ (38151)  2 sinusit*.tw. (16455)  3 rhinitis/ (15912)  4 rhinosinusitis/ (5169)  5 (rhinit* or rhinosinusit* or nasosinusit*).tw. (46826)  6 ((suppurative or purulent) adj2 ("nasal discharge" or rhinitis or rhinorrhea or rhinorrhoea)).tw. (417)  7 1 or 2 or 3 or 4 or 5 or 6 (80264)  8 exp antibiotic agent/ (1195320)  9 antibiotic.tw. (237108)  10 (Amoxicillin or ampicillin or azithromycin or cefaclor or cefadroxil or cefatrizine or cefuroxim* or cephalexin* or cephalosporin* or ciprofloxacin* or clarithromycin* or clindamycin).mp. (276181)  11 (doxycyclin* or erythromycin* or fluoroquinolone*).mp. (116781)  12 (levofloxacin* or lincomycin* or macrolide* or minocyclin* or miocamycin*).mp. (92691)  13 (miokamycin* or moxifloxacin* or norfloxacin* or ofloxacin* or penicillin*).mp. (133505)  14 (quinolone* or spiramycin or telithromycin or tetracyclin* or trimethoprim* or cotrimoxazol*).mp. (155867)  15 erythromycin*.mp. (54695)  16 8 or 9 or 10 or 11 or 12 or 13 or 14 or 15 (1329470)  17 7 and 16 (13855)  18 exp randomized controlled trial/ or exp single blind procedure/ or exp double blind procedure/ or exp crossover procedure/ (680624)  19 (crossover* or cross*over or "cross over" or placebo* or (doubl* adj1 blind*) or allocat* or random*).tw. (1743223)  20 trial.m_titl. (300714)  21 18 or 19 or 20 (1906850)  22 animal/ or nonhuman/ or animal experiment/ (6193415)  23 human/ (18062046)  24 22 and 23 (1935065)  25 22 not 24 (4258350)  26 21 not 24 (1832270)  27 17 and 26 (1346)  28 limit 27 to yr="2018 - 2020" (154) |

**Table S2. Characteristics of excluded studies**

| **Study** | **Year** | **Country** | **N** | **Antibiotic** | **Population** | **Age** | **Gender**  **(M:F, %)** | **Outcomes in the original study** |
| --- | --- | --- | --- | --- | --- | --- | --- | --- |
| Garbutt et al.^1^ | 2012 | USA | 166 | Amoxicillin | Patients presenting to primary care with moderate, severe, or very severe symptoms of suspected acute bacterial rhinosinusitis according to the Centers for Disease Control and Prevention’s expert panel’s diagnostic criteria | 18-70y | 60:106  (36:64%) | SNOT-16 scores at day 3 and symptom change since enrolment (0-6 Likert scale) |

; F = female; M = male; SNOT-16 = Sino-Nasal Outcome Test-16; USA = United States of America; y = years.

**Table S3. Characteristics of included studies**

| **Study** | **Year** | **Country** | **N** | **Antibiotic** | **Clinical diagnosis** | **Age** | **Time to outcome assessment (days)** | **Method of outcome assessment** |
| --- | --- | --- | --- | --- | --- | --- | --- | --- |
| Stalman et al.^2^ | 1997 | Netherlands | 192 | Doxycycline | At least two: PND, preceding cold, pain on bending | ≥15y | 10 | Diary |
| Kaiser et al.^3^ | 2001 | Switzerland | 269 | Azithromycin | URTI (common cold or sinusitis) | ≥18y | 8 | Clinical exam |
| De Sutter et al.^4^ | 2002 | Belgium | 416 | Amoxicillin | URTI and PND | ≥12y | 10 | Questionnaire, diary,  clinical exam* |
| Bucher et al.^5^ | 2003 | Switzerland | 252 | Amoxicillin clavulanate | PND and frontal or maxillary pain | ≥18y | 14 | Telephone |
| Varonen et al.^6^ | 2003 | Finland | 150 | Amoxicillin, phenoxymethylpenicillin,  or doxycycline | Clinical diagnosis of acute maxillary sinusitis | ≥18y | 14 | Telephone |
| Meltzer et al.^7^ | 2005 | International | 503 | Amoxicillin | Moderate symptom score (PND, postnasal drip, nasal congestion, sinus headache, facial pain) | ≥12y | 15 | Diary |
| Merenstein et al.^8^ | 2005 | USA | 135 | Amoxicillin | Symptoms for least 7 days and PND or pus in nasal cavity or unilateral facial pain | ≥18y | 14 | Telephone |
| Williamson et al.^9^ | 2007 | UK | 240 | Amoxicillin | At least two: PND, unilateral face pain, pus in nasal cavity | ≥16y | 10 | Diary |
| Schering-Plough et al.^10^ | NP | International | 485 | Amoxicillin | Moderate symptom score (PND, postnasal drip,  nasal congestion, sinus headache, facial pain) | ≥12y | 15 | Diary |

NP: not published, PND: purulent nasal discharge, UK = United Kngdom; URTI: upper respiratory tract infection, USA = United States of America; y: years.

*) All three sources were used to measure the outcome.

**Table S4. Percentage of missing values**

| **Trial** | **Bucher** | **De Sutter** | **Kaiser** | **Meltzer** | **Merenstein** | **Schering-Plough** | **Stalman** | **Varonen** | **Williamson1** | **Williamson2** |
| --- | --- | --- | --- | --- | --- | --- | --- | --- | --- | --- |
| Treatment assignment | *0* | *0* | *0* | *0* | *0* | *0* | *0* | *1.3* | *0* | *0* |
| Sex | *0* | *0* | *0* | *0* | *0* | *0* | *0* | *0* | *0* | *0* |
| Age | *0* | *0* | *0* | *0* | *0* | *0* | *0* | *0* | *0* | *0* |
| Preceding URTI | *100* | *10.9* | *100* | *100* | *100* | *100* | *0* | *2.7* | *16.4* | *17.5* |
| Symptom duration prior to enrolment | *0.4* | *3.3* | *0.4* | *0* | *0* | *0* | *0* | *100* | *17.2* | *18.4* |
| Pain on bending | *0.4* | *8.4* | *100* | *100* | *100* | *100* | *0* | *100* | *16.4* | *20.2* |
| Teeth pain | *100* | *9.5* | *0.4* | *100* | *100* | *100* | *0* | *0* | *16.4* | *19.3* |
| Unilateral facial pain | *0.4* | *8.7* | *0* | *100* | *0* | *100* | *0* | *2* | *16.4* | *18.4* |
| PNDsr | *0.4* | *16.3* | *0* | *0* | *0* | *28.6* | *0* | *0* | *19* | *18.4* |
| Symptom severity | *0.4* | *1.4* | *2.3* | *0* | *0* | *1.7* | *2.1* | *0* | *17.2* | *17.5* |
| Fever (>37.5 C) | *4.4* | *9* | *0.4* | *0* | *0* | *0* | *100* | *0.7* | *0* | *0* |
| PNDex | *0.4* | *0* | *0* | *0* | *0* | *0* | *0* | *2.7* | *0* | *0* |
| PPDex | *0.4* | *0* | *0.4* | *100* | *100* | *100* | *0* | *4* | *100* | *100* |
|  |  |  |  |  |  |  |  |  |  |  |
| Cure (%) | *0.4* | *2.7* | *1.1* | *0.8* | *14.1* | *0.9* | *2.1* | *4* | *12.1* | *11.4* |

URTI: upper respiratory tract infection; PNDsr: self-reported purulent nasal discharge, PNDex: purulent nasal discharge upon examination, PPDex: purulent pharyngeal discharge upon examination

| Trial | n | Cure (%) | LP mean | LP sd | Membership  C-statistic |
| --- | --- | --- | --- | --- | --- |
| *Bucher* | 251 | 0.69 | 0.8 | 0.39 | 0.88 |
| *Desutter* | 368 | 0.35 | -0.37 | 0.48 | 0.79 |
| *Kaiser* | 266 | 0.56 | -0.15 | 0.35 | 0.92 |
| *Meltzer* | 490 | 0.69 | 0.73 | 0.34 | 0.86 |
| *Merenstein* | 135 | 0.49 | 0.49 | 0.35 | 0.82 |
| *Schering-Plough* | 458 | 0.66 | 0.69 | 0.32 | 0.85 |
| *Stalman* | 191 | 0.65 | 0.70 | 0.40 | 0.86 |
| *Varonen* | 150 | 0.77 | 0.73 | 0.41 | 0.91 |
| *Williamson1* | 116 | 0.64 | 0.78 | 0.43 | 0.77 |
| *Williamson2* | 114 | 0.65 | 0.75 | 0.36 | 0.80 |

**Table S5. Between-study heterogeneity in predictor and outcome distributions**

Columns show the study label, the percentage cure, the mean and standard deviation of the LP (linear predictor) of the common prediction model, and the membership c-statistic.

**Table S6. IECV results for the main effects model after omission of Schering-Plough data**

|  | Bucher | De Sutter | Kaiser | Meltzer | Merenstein | Stalman | Varonen | Williamson1 | Williamson2 |
| --- | --- | --- | --- | --- | --- | --- | --- | --- | --- |
| *C statistic* | 0.60 | 0.55 | 0.55 | 0.61 | 0.56 | 0.59 | 0.61 | 0.58 | 0.62 |
| *R^2^* | 0.03 | -0.47 | -0.57 | -4.96 | -0.29 | 0.03 | -0.24 | 0.01 | 0.05 |
| *Brier* | 0.21 | 0.30 | 0.32 | 0.50 | 0.29 | 0.22 | 0.21 | 0.23 | 0.22 |
| *Intercept* | 0.10 | -0.82 | 0.67 | 3.18 | -0.45 | -0.03 | 0.92 | 0.14 | -0.11 |
| *Slope* | 0.82 | 0.41 | 0.45 | 1.31 | 0.48 | 0.87 | 0.99 | 0.62 | 1.10 |

**Figure S1. Risk of bias assessment**


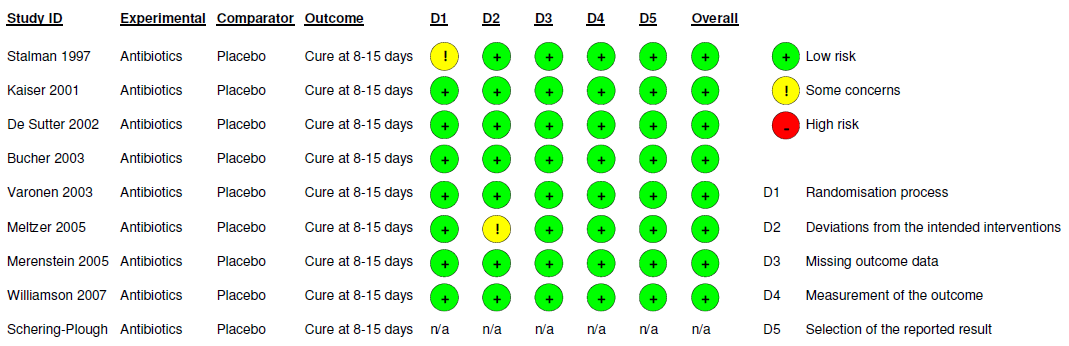


n/a= risk of bias assessment could not be performed for the unpublished Schering-Plough trial.

**Figure S2. Visual representation of the descriptive statistics across studies**

Boxplots for continuous variables; barplots for dichotomous variables. Trial numbers correspond to: (1) Merenstein, (2) Kaiser, (3) Stalman, (4) De Sutter, (5) Bucher, (6) Varonen, (7) Williamson1, (8) Williamson2, (9) Meltzer, and (10) Schering-Plough.

**Figure S3. Expected versus observed percentage cure in the context of the study of case-mix heterogeneity between studies**

The expected percentage cure was based on a fixed effects model with a common intercept and common main effects for each of the predictors. Related estimated are available in Table S5. Trial numbers correspond to: (1) Merenstein, (2) Kaiser, (3) Stalman, (4) De Sutter, (5) Bucher, (6) Varonen, (7) Williamson1, (8) Williamson2, (9) Meltzer, and (10) Schering-Plough.

**Figure S4. Boxplots of the distribution of random intercept estimates for each of the ten studies.**

Each box consists of 50 estimates corresponding to 50 imputed data sets. Study numbers correspond to: (1) Merenstein, (2) Kaiser, (3) Stalman, (4) De Sutter, (5) Bucher, (6) Varonen, (7) Williamson1, (8) Williamson2, (9) Meltzer, and (10) Schering-Plough..

**Figure S5. Internal-external cross-validation (IECV) of predicted of risk of cure (main analysis model)**

**Figure S5a Meta-analysis of the IECV c-statistics.**

**Figure S5b Meta-analysis of the IECV R-squared estimates.**

**Figure S5c Meta-analysis of the IECV Brier score estimates.**

**Figure S5d Meta-analysis of the IECV intercept estimates.**

**Figure S5e Meta-analysis of the IECV slope estimates.**

**Figure S6. Internal-external cross-validation (IECV) of discriminative performance (c-for-benefit) of predicted of individualized treatment effect (main analysis model)**

**Supplementary material 1**

**Heterogeneity in case-mix**

To provide further insight into the between-study case-mix heterogeneity, we follow guidance by Steyerberg et al.^11^ Thereto, a logistic prediction model for the outcome and predictors was derived with common coefficients (*i.e.* the same coefficients for each study). The mean and standard deviation of the estimated linear predictor values reflect the overall effect of case-mix heterogeneity as weighted by the model coefficients (Table S5).

With respect to the interpretation, the degree of variability of the individual linear predictor estimates (sd) reflects within study variability in case-mix. For instance, study by De Sutter et al.^4^ has the highest within study case-mix variability as weighted by the model coefficients.

The estimated distribution of the linear predictor also provides insight into the percentage cure that should be expected for a particular study based on the common model. Supplementary Figure 3 shows the observed versus expected percentage cure for each study, and shows that four studies (Merenstein et al.^8^, Kaiser et al. ^3^, de Sutter et al. ^4^, and Varonen et al. ^6^) deviate somewhat from the expectations. This shows that the effect of case-mix as modeled by the common main effects does not fully explain the heterogeneity in cure incidence across studies. In the main analysis, such variability is captured by the random intercepts (Supplementary Figure S4).

In line with Steyerberg et al.^11^, a multinomial membership model was derived in order to predict individual study membership based on the predictors of interest and the observed outcomes. More specifically, a multinomial ridge regression model was derived with study membership as the dependent variable, main effect for the outcome and predictor variables, and a 10-fold cross-validation-based penalty parameter estimate. A pooled model was derived based on the average coefficients across imputations. Performance of this model was subsequently pooled across imputations. Table S5 shows the c-statistic describing the discriminative ability. The latter was measured dichotomously per study and hence describes discriminative between a particular study and all other studies. The estimated c-statistics were mostly high, indicating large between-study variability (*i.e.,* easy separation between studies).

**References**

1. Garbutt JM, Banister C, Spitznagel E, Piccirillo JF. Amoxicillin for Acute Rhinosinusitis: A Randomized Controlled Trial. *JAMA*. 2012;307(7):685. doi:10.1001/jama.2012.138

2. Stalman W, Melker RAD. The end of antibiotic treatment in adults with acute sinusitis-like complaints in general practice? A placebo-controlled double-blind randomized doxycycline trial. *British Journal of General Practice*. Published online 1997:6.

3. Kaiser L, Morabia A, Stalder H, et al. Role of Nasopharyngeal Culture in Antibiotic Prescription for Patients with Common Cold or Acute Sinusitis. *European Journal of Clinical Microbiology and Infections Diseases*. 2001;20(7):0445-0451. doi:10.1007/s100960100544

4. De Sutter AI, De Meyere MJ, Christiaens TC, Van Driel ML, Peersman W, De Maeseneer JM. Does amoxicillin improve outcomes in patients with purulent rhinorrhea? A pragmatic randomized double-blind controlled trial in family practice. *J Fam Pract*. 2002;51(4):317-323.

5. Bucher HC. Effect of Amoxicillin-Clavulanate in Clinically Diagnosed Acute Rhinosinusitis: A Placebo-Controlled, Double-blind, Randomized Trial in General Practice. *Arch Intern Med*. 2003;163(15):1793. doi:10.1001/archinte.163.15.1793

6. Varonen H, Kunnamo I, Savolainen S, et al. Treatment of acute rhinosinusitis diagnosed by clinical criteria or ultrasound in primary care. *Scandinavian Journal of Primary Health Care*. 2003;21(2):121-126. doi:10.1080/02813430310001743

7. Meltzer E, Bachert C, Staudinger H. Treating acute rhinosinusitis: Comparing efficacy and safety of mometasone furoate nasal spray, amoxicillin, and placebo. *Journal of Allergy and Clinical Immunology*. 2005;116(6):1289-1295. doi:10.1016/j.jaci.2005.08.044

8. Merenstein D, Whittaker C, Chadwell T, Wegner B, D’Amico F. Are antibiotics beneficial for patients with sinusitis complaints? A randomized double-blind clinical trial. *J Fam Pract*. 2005;54(2):144-151.

9. Williamson IG, Rumsby K, Benge S, et al. Antibiotics and Topical Nasal Steroid for Treatment of Acute Maxillary Sinusitis: A Randomized Controlled Trial. *JAMA*. 2007;298(21):2487. doi:10.1001/jama.298.21.2487

10. Schering-Plough Research Institute. *Efficacy and Safety of 200 Mcg QD or 200 Mcg BID Mometasone Fuorate (MFNS) vs Amoxicillin vs Placebo as Primary Treatment of Subjects with Acute Rhinosinusitis (Protocol P02692)*. Schering-Plough Research Institute,; 2003.

11. Steyerberg EW, Nieboer D, Debray TPA, Houwelingen HC. Assessment of heterogeneity in an individual participant data meta‐analysis of prediction models: An overview and illustration. *Statistics in Medicine*. 2019;38(22):4290-4309. doi:10.1002/sim.8296
